# Integrated analysis of transcriptome and metabolites reveals an essential role of metabolic flux in starch accumulation under nitrogen starvation in duckweed

**DOI:** 10.1186/s13068-017-0851-8

**Published:** 2017-06-26

**Authors:** Changjiang Yu, Xiaowen Zhao, Guang Qi, Zetao Bai, Yu Wang, Shumin Wang, Yubin Ma, Qian Liu, Ruibo Hu, Gongke Zhou

**Affiliations:** 1 Shandong Provincial Key Laboratory of Energy Genetics, Qingdao Engineering Research Center of Biomass Resources and Environment, Qingdao Institute of Bioenergy and Bioprocess Technology, Chinese Academy of Sciences, Qingdao, 266101 People’s Republic of China; 2 University of Chinese Academy of Sciences, Beijing, 100049 People’s Republic of China; 3 College of Life Sciences, China Agricultural University, Beijing, 100094 People’s Republic of China; 4 Guangzhou Genedenovo Biotechnology Co., Ltd, Guangzhou, 510006 China

**Keywords:** Duckweed, Transcriptome sequencing, Nitrogen stress, Metabolic flux, Starch accumulation

## Abstract

**Background:**

Duckweed is considered a promising source of energy due to its high starch content and rapid growth rate. Starch accumulation in duckweed involves complex processes that depend on the balanced expression of genes controlled by various environmental and endogenous factors. Previous studies showed that nitrogen starvation induces a global stress response and results in the accumulation of starch in duckweed. However, relatively little is known about the mechanisms underlying the regulation of starch accumulation under conditions of nitrogen starvation.

**Results:**

In this study, we used next-generation sequencing technology to examine the transcriptome responses of *Lemna aequinoctialis* 6000 at three stages (0, 3, and 7 days) during nitrogen starvation in the presence of exogenously applied sucrose. Overall, 2522, 628, and 1832 differentially expressed unigenes (DEGs) were discovered for the treated and control samples. Clustering and enrichment analysis of DEGs revealed several biological processes occurring under nitrogen starvation. Genes involved in nitrogen metabolism showed the earliest responses to nitrogen starvation, whereas genes involved in carbohydrate biosynthesis were responded subsequently. The expression of genes encoding nitrate reductase, glutamine synthetase, and glutamate synthase was down-regulated under nitrogen starvation. The expression of unigenes encoding enzymes involved in gluconeogenesis was up-regulated, while the majority of unigenes involved in glycolysis were down-regulated. The metabolite results showed that more ADP-Glc was accumulated and lower levels of UDP-Glc were accumulated under nitrogen starvation, the activity of AGPase was significantly increased while the activity of UGPase was dramatically decreased. These changes in metabolite levels under nitrogen starvation are roughly consistent with the gene expression changes in the transcriptome.

**Conclusions:**

Based on these results, it can be concluded that the increase of ADP-glucose and starch contents under nitrogen starvation is a consequence of increased output from the gluconeogenesis and TCA pathways, accompanied with the reduction of lipids and pectin biosynthesis. The results provide novel insights into the underlying mechanisms of starch accumulation during nitrogen starvation, which provide a foundation for the improvement of advanced bioethanol production in duckweed.

**Electronic supplementary material:**

The online version of this article (doi:10.1186/s13068-017-0851-8) contains supplementary material, which is available to authorized users.

## Background

With increasing dependence on fossil fuels, which is unsustainable and has increasingly high environmental, economic, and political costs, there has been a push to identify alternative renewable energy sources [1, 2]. Ethanol production from dedicated bioenergy crops or agricultural residues is one of the more promising technologies that can help address this pressing issue. Corn is currently the predominant feedstock for ethanol production in China. However, corn-based ethanol production endangers domestic food security because it competes with food and feed production due to limited agricultural land [3]. Therefore, there is an urgent need to explore alternative feedstock sources for ethanol production [4].

Species of the *Lemnaceae* family (duckweed) are the world’s smallest, but fastest growing, aquatic flowering plants. *Lemnaceae* is a monocotyledonous family with 37 species in five genera: *Spirodela, Landoltia, Lemna, Wolffia*, and *Wolffiella* [5]. Duckweed is commonly used for bioremediation of nutrient-rich wastewater and as fodder for fish and ducks due to its ability to absorb substantial amounts of nutrients and high protein content. However, recent research has demonstrated that duckweed can also be regarded as a potential feedstock for bioethanol production due to its impressive ability for starch accumulation [6–8]. The starch content of duckweed varies significantly (dry weight) depending on the species and growing conditions [4, 9]. The effects of various stress conditions, such as nitrogen starvation, temperature extremes, and salt, on starch accumulation in duckweed have been addressed [8, 10]. However, compared to a model plant (i.e., *Arabidopsis thaliana*), the molecular mechanism of starch accumulation during physiological stress remains largely unknown in other plant species, especially in duckweed. Although the genome sequences of duckweed species (*Spirodela polyrhiza* and *Lemna minor*) have been released, the availability of the genomic sequences is somewhat restricted because the genome sizes vary enormously, ranging from 150 Mb in *Spirodela* to 1881 Mb in *Wolffia*, which is a 13-fold difference [11]. However, with the rapid evolution of next-generation sequencing strategies and decreases in sequencing costs, transcriptome sequencing serves as an alternative and more efficient approach because it targets only coding DNA, which can facilitate the de novo assembly of transcriptomes from non-model species [12, 13].

To date, three studies of transcriptome analysis in duckweed species have been published. A study in *Landoltia punctate* showed that the gene expression level of starch degradation genes were negatively correlated with biosynthesis genes, which led to starch accumulation [14]. Another RNA-Seq study focused on the transcriptome analysis of *Spirodela* dormancy without reproduction and showed that key genes involved in starch synthesis (e.g., *APS1*, *APL3,* and *GBSSI*) were triggered by the application of exogenous ABA [15]. A third RNA-seq analysis revealed dose rate-dependent gene expression responses and indicated that *Lemna minor* shifts from acclimation responses towards survival responses at increasing dose rates of ionizing radiation [16]. Currently, the molecular mechanism of a single stressor on starch accumulation in duckweed is yet to be revealed. In the current study, we performed a transcriptome survey to reveal the molecular mechanism of starch accumulation under nitrogen starvation in *L. aequinoctialis* 6000, which is a fast-growing and high starch species of duckweed.

In this study, we analyzed the transcriptomic profiles and metabolite patterns of duckweed (*Lemna aequinoctialis* 6000) during a 7-day time course under nitrogen starvation. Differentially expressed genes (DEGs) were identified and categorized as various functional classifications and nitrogen starvation-related metabolic metabolisms, which were further validated by examining specific enzyme activities. The results demonstrate the feasibility of using transcriptome data with a next-generation sequencing (NGS) method to identify interesting pathways and potential target genes, which could serve as excellent candidates for functional genomics studies and metabolic engineering to improve the production of next-generation biofuels in duckweed.

## Methods

### Plant materials and growth conditions

*Lemna aequinoctialis* 6000 was obtained from a screening of more than 100 duckweed species distributed across 20 provinces in China [17]. The strain used in this study was collected from Li Xian in Hunan Province. The plants were cultured in a growth chamber at 24 °C under a 16 h light/8 h dark photoperiod and at a light intensity of 110 µmol m^−2^ s^−1^ provided by wide spectrum fluorescent tubes. The light intensity was measured using a quantum photometer (LI-250A, LI-COR, USA). The plants were grown in liquid Schenk and Hildebrandt (SH) medium supplemented with 10 g/L sucrose for 2 weeks, then transferred to liquid SH medium that lacked nitrogen but was also supplemented with 10 g/L sucrose. Samples were collected at three different time points (0, 3, and 7 days) at 2 pm for the RNA-Seq analysis. The sampling time points were determined based on a pilot experiment that examined the expression of the ADP-glucose pyrophosphorylase gene, which is an important marker gene in starch biosynthesis [18].

### Starch content and periodic acid–Schiff (PAS) staining

Starch granules were stained using the Periodic Acid–Schiff (PAS) Kit (Sigma-Aldrich) according to the manufacturer’s instructions. Total starch content was determined using a Megazyme total starch assay kit. Briefly, 100 mg of frozen dry duckweed was ground into powder with a multi-tube ball mill (Tissuelyser II, Qiagen, Germany) and transferred into a test tube. To aid dispersion, 0.2 mL of 80% ethanol was stirred into the sample with a vortex mixer. The tube was then placed in a 50° water bath, and 4 mL of sodium acetate was added, followed by the addition of ~3 units of thermo-stable alpha-amylase (Sigma A4582, USA) and ~6 units amyloglucosidase (Sigma A7095, USA). The sample tube was then agitated with a vortex mixer and incubated for 30 min in a 50 °C water bath. The contents were then moved/washed into a 100 mL volumetric flask with distilled water, mixed, and centrifuged at 3000 rpm for 10 min. A 0.1 mL aliquot of the supernatant was added to 2 mL of GOPOD reagent, vortexed, and incubated at 50 °C for 20 min. Blank (0.1 mL of distilled water and 2 mL of GOPOD) and glucose standards (0.2, 0.4, 0.6, 0.8, and 1.0 mg/mL) were incubated concurrently. Absorbance was measured with a spectrophotometer at 510 nm with a path length of 1 cm.

### RNA extraction and library construction

Total RNA was extracted with Trizol reagent (Invitrogen, USA) and then treated with RNase-Free DNase I (TaKaRa, Japan) to remove DNA contaminants. RNA integrity was confirmed using agarose gel electrophoresis and an Agilent 2100 Bioanalyzer (Agilent, USA). RNA concentrations were determined using a Qubit 2.0 spectrophotometer (Life Technology, USA). For each treatment, mRNA was purified from 10 µg of total RNA using magnetic oligo (dT) beads and fragmented using fragmentation buffer with the preparation kit according to the manufacturer’s instructions (Invitrogen, USA). The cleaved short RNA fragments were used for first-strand cDNA synthesis using reverse transcriptase and hexamer primer (Illumina), and the second strand cDNA was synthesized with DNA polymerase I and RNase H (Thermo Fisher, USA). cDNA fragments were selected for PCR amplification, and cDNA libraries were used for sequencing with the Illumina HiSeq™ 2000 platform.

### Data filtering and de novo assembly

Raw reads obtained after sequencing were firstly filtered by removing reads containing adapters, reads containing more than 10% of unknown nucleotides (N), and low quality reads containing more than 50% of low quality (*Q* < 20) bases using custom Perl scripts. The clean reads were then assembled into unique consensus sequences using Trinity [19]. The redundancy was eliminated using TGICL [20] and further assembled into a single set of non-redundant unigenes.

### Functional annotation of unigenes

Six databases were used for unigene annotation, including the NCBI protein database (Nr), the NCBI nucleotide database (Nt), the euKaryotic Orthologous Groups database (KOG), the Swiss-Prot database, the KEGG Orthology database, and the Gene Ontology (GO) databases. These annotations were performed using a combination of BLAST, Blast2GO, HMMER, and KEGG Automatic Annotation Server (KAAS) tools.

### Analysis of differential expression genes

Using the transcriptome assembled by Trinity [19] as a reference, clean reads of each sample were mapped by RSEM [21]. The normalized read counts from two replicates of each sample were analyzed, and unigenes showing significant differences in expression were determined using DEseq [22]. The FDR* q* value threshold was set to 0.005, and the fold change of expression was set to 2.0.

### Gene ontology and Kyoto Encyclopedia of Genes and Genomes (KEGG) enrichment analysis

Gene ontology and KEGG enrichment analysis was carried out to determine the distributions of unigenes based on their functions and biological pathways. GO analysis was performed using Goseq based on Wallenius’ non-central hypergeometric distributions [23]. KEGG enrichment analysis was performed using KOBAS 2.0 with hypergeometric tests [24]. GO categories and KEGG pathways with FDR *q* value ≤0.05 were considered to be significantly enriched.

### Quantitative real-time RT-PCR

Quantitative real-time RT-PCR (qRT-PCR) was performed to validate the expression of unigenes. Gene-specific primers for seven randomly selected DEGs were designed by Beacon Designer 8.0. The 18S gene was used as the internal control. First-strand cDNA was synthesized using TransScript One-Step gDNA Removal and cDNA Synthesis SuperMix (TransGen Biotech, China) according to the manufacturer’s instructions. Reactions were carried out using a SYBR Premix Ex Taq II (Tli RnaseH Plus) Kit (TaKaRa, Japan) in a Lightcycler 480 Real-Time PCR Detection System (Roche, Germany). Amplification reaction mixtures consisted of 10 µL of SYBR Premix Ex Taq II, 0.2 µL of each forward and reverse primer (10 mM), and 1 µL of cDNA template, and sterile water was added to a final volume of 20 µL. The amplification cycling program was as follows: 94 °C for 30 s, 42 cycles of 94 °C for 5 s, and 59 °C for 30 s. The relative expression was calculated using the 2^−ΔΔCT^ method [25]. qRT-PCR analysis was conducted with three technical replicates, and the data represent the means ± standard errors (*n* = 3).

### Carbohydrate measurement and enzyme activity assays

Leaves were flash frozen, then extracted in chloroform/methanol. Starch, soluble glucans, and sugars were extracted and assayed by enzymatic methods [26]. To examine the enzyme activities, 1 g of fresh duckweed was homogenized with a ceramic pestle in an ice-cold mortar in 5 mL of reaction solution [50 mM HEPES–NaOH (pH 7.6), 5 mM dl-dithiothreitol, 8 mM MgCl_2_, 2 mM EDTA, 2% (w/v) PVP-40, and 12.5% (w/v) glycerol]. The homogenate was centrifuged at 10,000×*g* for 5 min. The supernatant was used as a crude enzyme solution and stored at −20 °C. The activities of α-amylase (1,4, d-glucan glucanohydrolase, 3.2.1.1) and β-amylase (1,4, d-glucan maltohydrolase, 3.1.3.12) were determined following the method of Tarrago and Nicolas [27]. The enzymatic activities of soluble starch synthases (SSs, 2.4.1.21) and AGPase (2.7.7.9) were assayed according to Nakamura et al. [28]. Starch synthase activity was assayed according to Jenner et al. [29]. To visualize glycogen and starch synthase activities, leaves were extracted with 100 mM MOPS (pH 7.2), 1 mM EDTA, 1 mM DTT, and 10% (w/v) glycerol (300 mg sample/mL). Extracts were loaded onto non-denaturing polyacrylamide gels containing 0.3% (w/v) glycogen. After incubation for 16 h at 20 °C in 100 mM HEPES–NaOH (pH 7.5), 2 mM DTT, 10% (v/v) glycerol, 0.5 mM EDTA, 0.5 M sodium citrate, and 2 mM ADP-glucose, both starch and glycogen synthase activities were revealed by iodine staining.

## Results

### Impact of nitrogen starvation in the presence of exogenously applied sucrose on starch accumulation

**Fig. 1:**
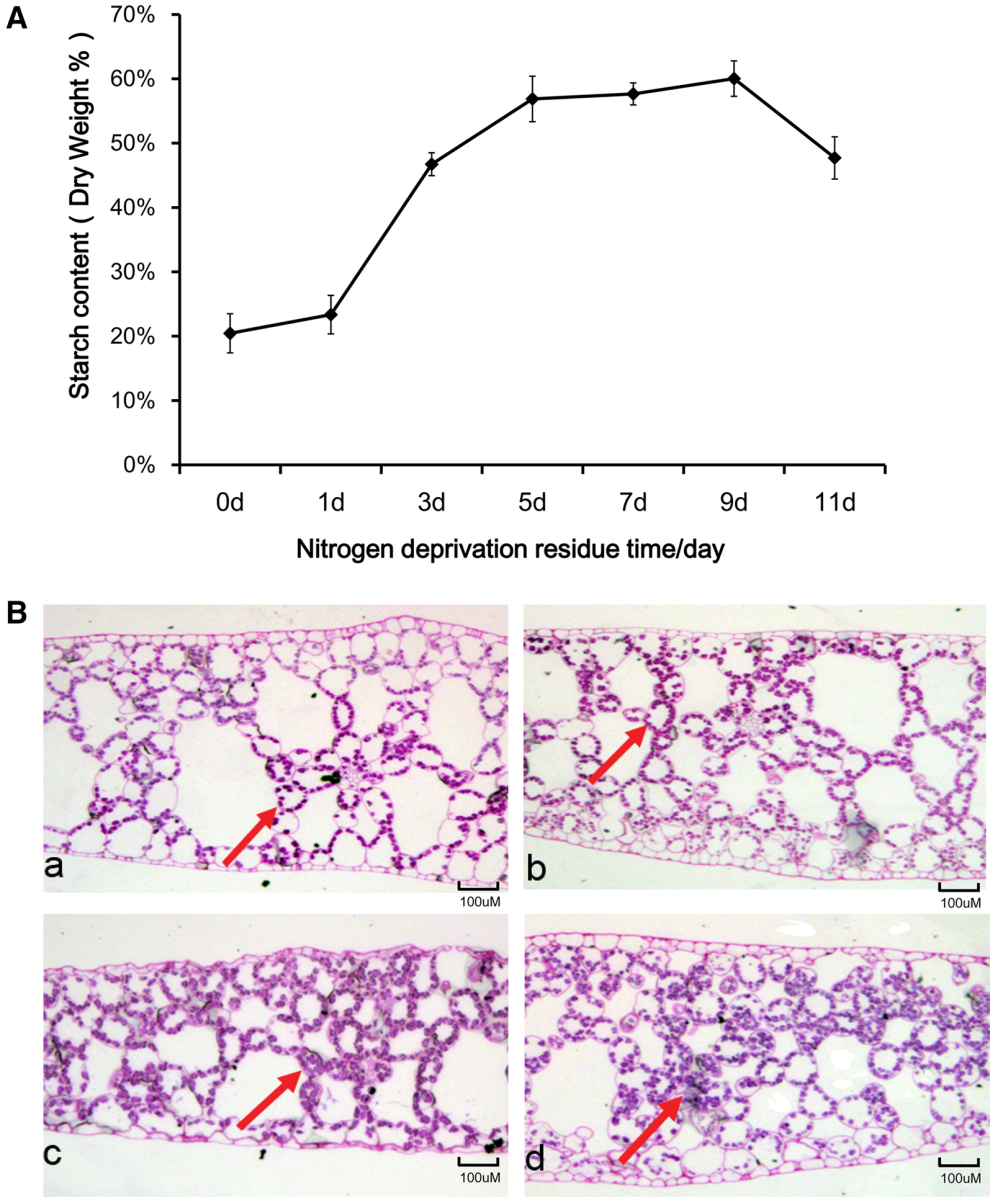
Starch accumulation in *L. aequinoctialis* 6000 during nitrogen starvation. **A** Quantification of starch contents at different stages of nitrogen starvation. **B** Cross sections of duckweed fronds showing accumulation of starch granules during nitrogen starvation.** a**–**d** indicate 0 day (**a**), 3 days (**b**), 5 days (**c**), and 7 days (**d**) during nitrogen starvation. *Red arrows* indicate the starch granules stained with periodic acid–Schiff (PAS)

The starch content of *L. aequinoctialis* 6000 grown under nitrogen starvation conditions was measured at three different time points. The initial starch content was measured at approximately 20% before treatment and then increased rapidly over the following 9 days during nitrogen starvation (Fig. 1A). The highest starch content was 60 ± 3% after treatment for 9 days, which was almost three times higher compared to the pre-treatment. The results showed that *L. aequinoctialis* had considerable starch accumulation during nitrogen starvation. Therefore, this plant can be used as an ideal experimental model to characterize the mechanisms underlying starch accumulation. We further determined the starch granule content through in situ staining of fronds with periodic acid–Schiff (PAS) (Fig. 1B). The results indicated that the number of starch granules significantly increased under nitrogen starvation, which was consistent with the results in Fig. 1A. The starch granules were sparsely distributed in fronds at the cell edge (Fig. 1B (a)) and then gradually accumulated to the cell center (Fig. 1B (b–d)) as the starch content increased. The starch granules occupied almost the entire cell after 7 days of nitrogen starvation (Fig. 1B (d)).

### Illumina HiSeq mRNA sequencing

To identify the genes responsible for starch accumulation, we explored the transcriptome profile of *L. aequinoctialis* 6000 during nitrogen starvation using RNA-seq. Overall, 25 G sequencing data were generated and de novo assembled into 72,105 unigenes with an average length of 1233 bp (Table 1; Additional file 1: Table S1; Additional file 2: Table S2).

**Table 1:** Assembly quality statistics of the *L. aequinoctialis* transcriptome

Samples	Total reads	Total unigene number	Total length (nt)	Mean length (nt)	N50	Total consensus sequence	Distinct clusters	Distinct singletons
0d-1	45,631,688	64,360	56,557,875	879	1687	64,360	35,175	29,185
0d-2	42,041,558	51,699	40,541,311	784	1453	51,699	28,052	23,647
3d-1	33,961,764	51,664	42,605,247	825	1559	51,664	27,845	23,819
3d-2	33,586,294	51,250	40,967,920	799	1523	51,250	27,545	23,705
7d-1	46,889,100	58,605	52,433,518	895	1681	58,605	33,003	25,602
7d-2	44,459,216	50,940	41,122,923	807	1539	50,940	27,090	23,850
All		72,105	88,895,700	1233	1916	72,105	47,937	24,168

Functional annotation of the unigenes was carried out in a BLAST search of public databases, including the NCBI Nr and Nt database, the GO database, the KOG database, the Swiss-Prot protein database, and the KEGG database. Overall, 53,734 (74.52%) unigenes were functionally annotated according to their similarities with known genes/proteins in these databases. The detailed statistics of the functional annotation are provided in Additional file 3: Figure S1.

### Identification of differentially expressed genes during nitrogen starvation

**Fig. 2:**
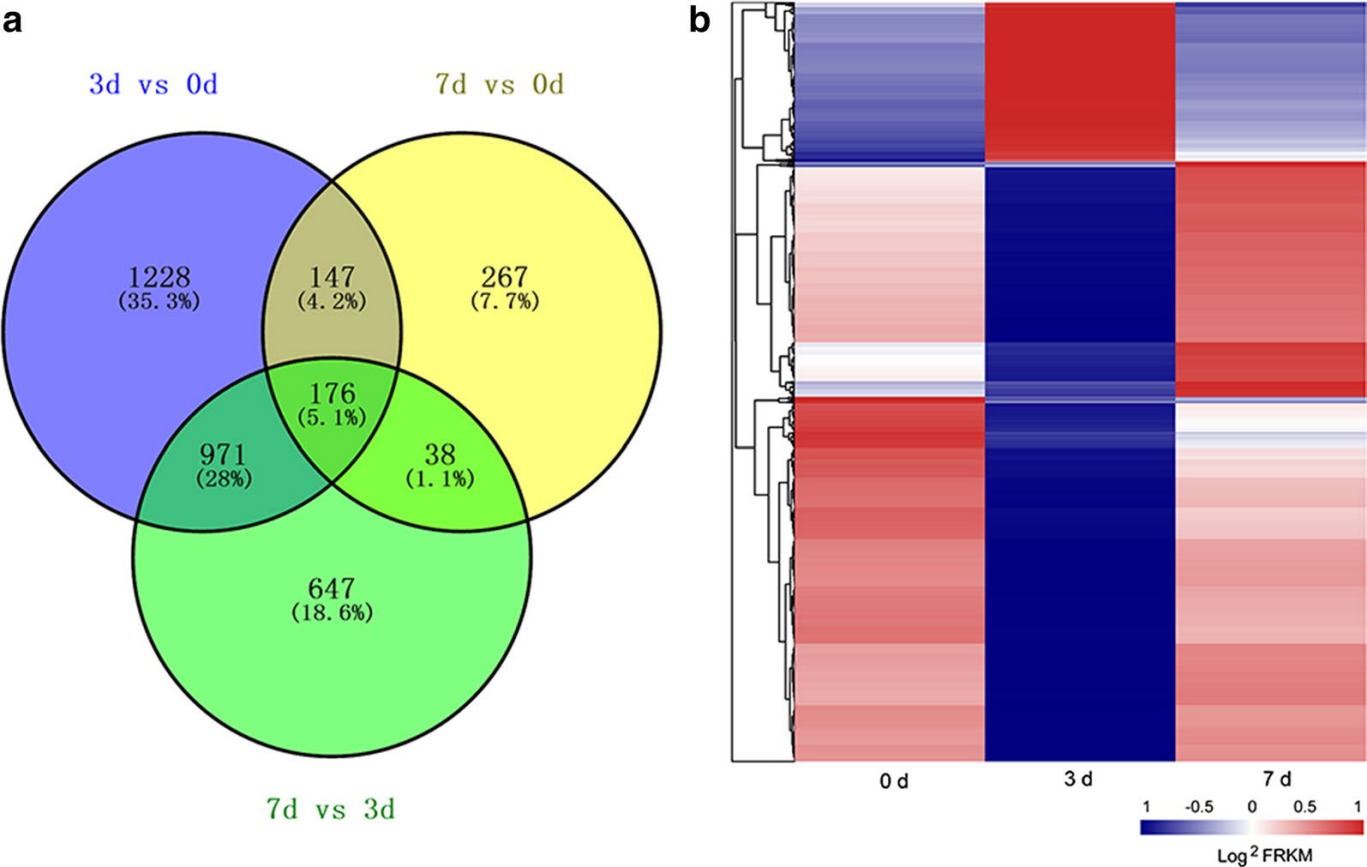
Statistics of the differentially expressed genes in *L. aequinoctialis* during nitrogen starvation. **a** Venn diagram showing differentially expressed genes at 3 and 7 days after nitrogen starvation compared to the 0 day control; **b** hierarchical clustering of all differentially expressed genes (DEGs) based on normalized FPKM values in three time points. *Blue* indicates lower expression, and *red* indicates higher expression

The expression profiles of *L. aequinoctialis* cultivated under nitrogen starvation conditions were analyzed. Compared to the samples at 0 day, the expression of 2522 unigenes changed after nitrogen starvation for 3 days, whereas the expression of 628 unigenes were affected at 7 days under nitrogen starvation. There were 323 differentially expressed genes (DEGs) in both the 3- and 7-day treatments (Fig. 2a). All DEGs were subjected to GO term analysis (Additional file 4: Figure S2), KOG analysis (Additional file 5: Figure S3), and KEGG pathway enrichment analysis (Additional file 6: Figure S4). There were 157,910 (48.66%) unigenes assigned to a biological process, 123,574 (38.08%) unigenes assigned to a cellular component, and 43,007 (13.25%) unigenes assigned to a molecular function. Overall, 35,529 unigenes (49.27%) were classified into 25 KOG categories. Among the 25 KOG categories, the “cluster of general function prediction” (9560, 26.91%) represented the largest group, followed by “transcription” (5851, 16.47%), “replication, recombination, and repair” (5089, 14.32%), “signal transduction mechanisms” (4359, 12.26%), “translation, ribosomal structure, and biogenesis” (3521, 9.91%), and “carbohydrate transport and metabolism” (3461, 9.74%). A total of 35,529 (49.27%) unigenes had hits for 128 KEGG pathways. “Metabolic pathways” contained the most unigenes (8546, 24.05%), followed by “Biosynthesis of secondary metabolites” (3719, 10.47%), “RNA transport” (2330, 6.56%), and “Spliceosome” (2119, 5.96%). The remaining 117 KEGG pathways each contained less than 1000 unigenes.

**Fig. 3:**
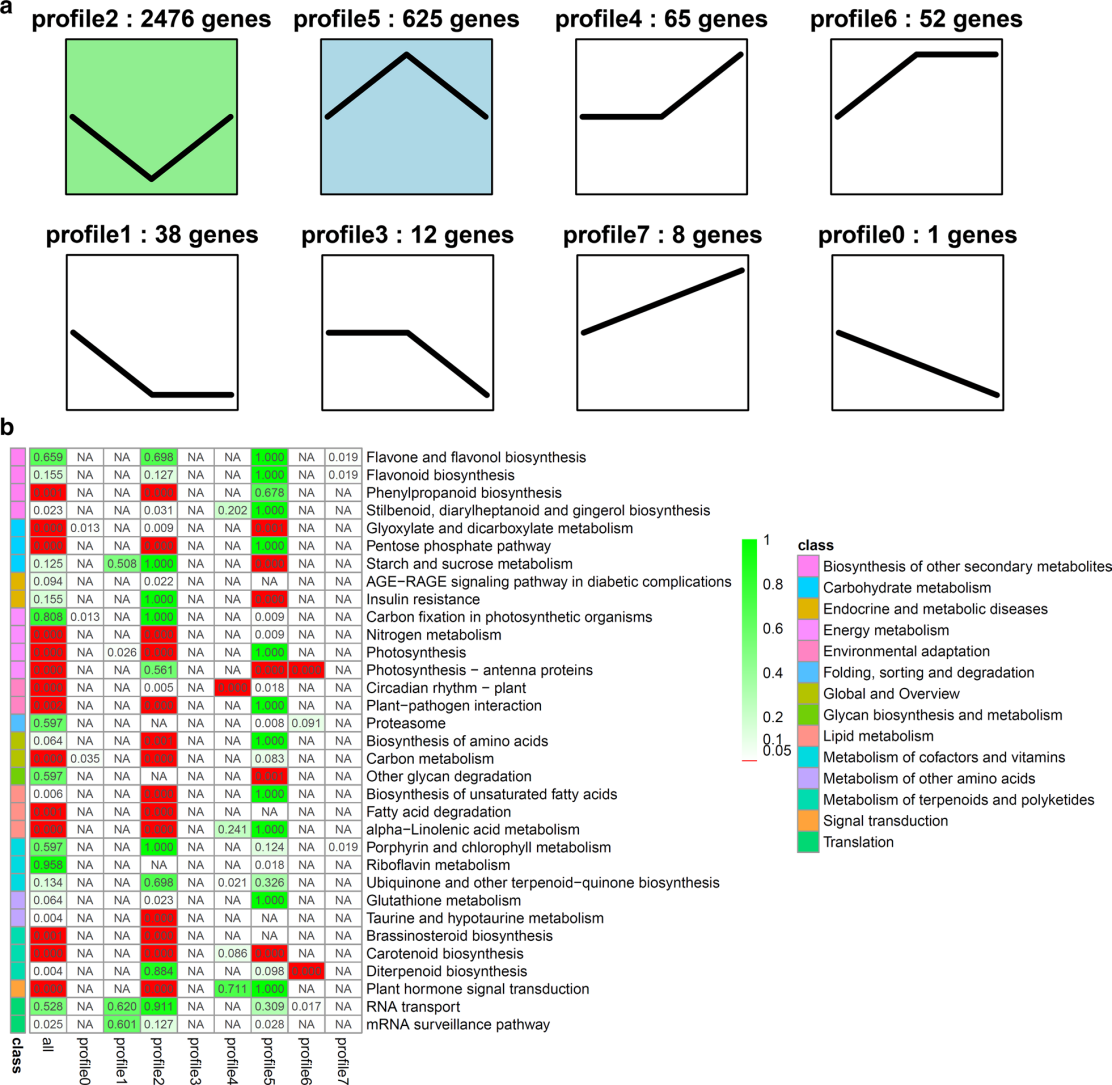
Dynamic transcriptome of *L. aequinoctialis* during nitrogen starvation. **a** Cluster of gene expression patterns in response to nitrogen starvation. Short time-series expression miner (STEM) was used to analyze the gene expression pattern, and eight profiles exhibited significant clustering of gene expression patterns. The number of unigenes in each profile was labeled above the frame. The *black line* represents the general tendency in each profile. Expression patterns of 8 profiles at different stages under nitrogen starvation. **b** Enrichment of functional categories of each cluster with the significantly enriched KEGG pathways plotted for DEGs among the three stages of nitrogen starvation

To further explore the biological function of these DEGs, hierarchical clustering was performed using the Euclidean distance associated with complete-linkage analysis (Fig. 2b). Expression pattern analysis was also carried out, and a total of eight clusters were identified (cluster 0–cluster 7, Fig. 3). The enrichment analysis was performed for each profile to identify the putative pathways associated with starch accumulation under nitrogen starvation (Additional file 7: Figure S5, Additional file 8: Figure S6).

As shown in Fig. 3, the genes in cluster 2 and cluster 5 represented two of the largest of DEGs with 2476 and 625 genes, respectively, and the genes in these two clusters had opposite expression patterns. The genes in cluster 2 were significantly down-regulated at 3 days compared to the 0 and 7 days levels during nitrogen starvation. In contrast, the genes in cluster 5 were significantly up-regulated at 3 days compared to the other time points during nitrogen starvation. Because cluster 2 and cluster 5 represented two of the largest groups of DEGs, we conducted an enrichment analysis of functional categories for DEGs in these two clusters. The significantly enriched categories in cluster 2 included nitrogen metabolism-related pathways as well as pathways involved in the light reaction of photosynthesis, carotenoid biosynthesis, amino acid biosynthesis, carbon metabolism, and unsaturated fatty acid biosynthesis. The enriched categories in cluster 5 included starch and sugar metabolism, glycan degradation, glyoxylate and dicarboxylate metabolism, and riboflavin metabolism.

### Pathways induced by nitrogen starvation

**Fig. 4:**
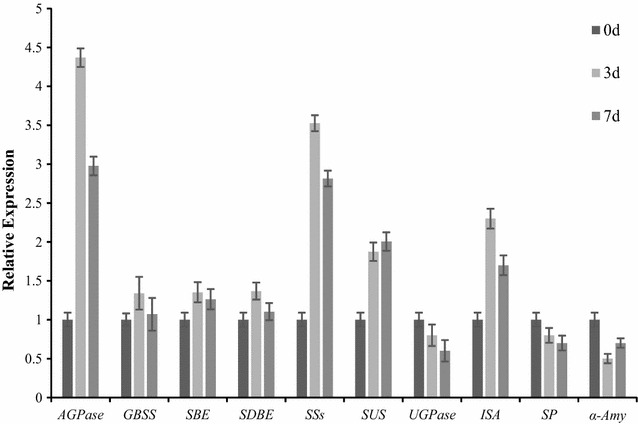
Validation of the expression of candidate genes during nitrogen starvation by quantitative Real-time RT-PCR analysis. The expression at 0 day was set as 1, and the relative expression level was calculated for several genes. *AGPase* ADP-glucose pyrophosphorylase, *GBSS* granule bound starch synthase, *SBE* starch-branching enzyme, *SDBE* starch-debranching enzyme, *SSs* soluble starch synthase, *SUS* sucrose synthase, *UGPase* UDP-glucose pyrophosphorylase, *ISA* isoamylase, *SP* sucrose phosphorylase

**Fig. 5:**
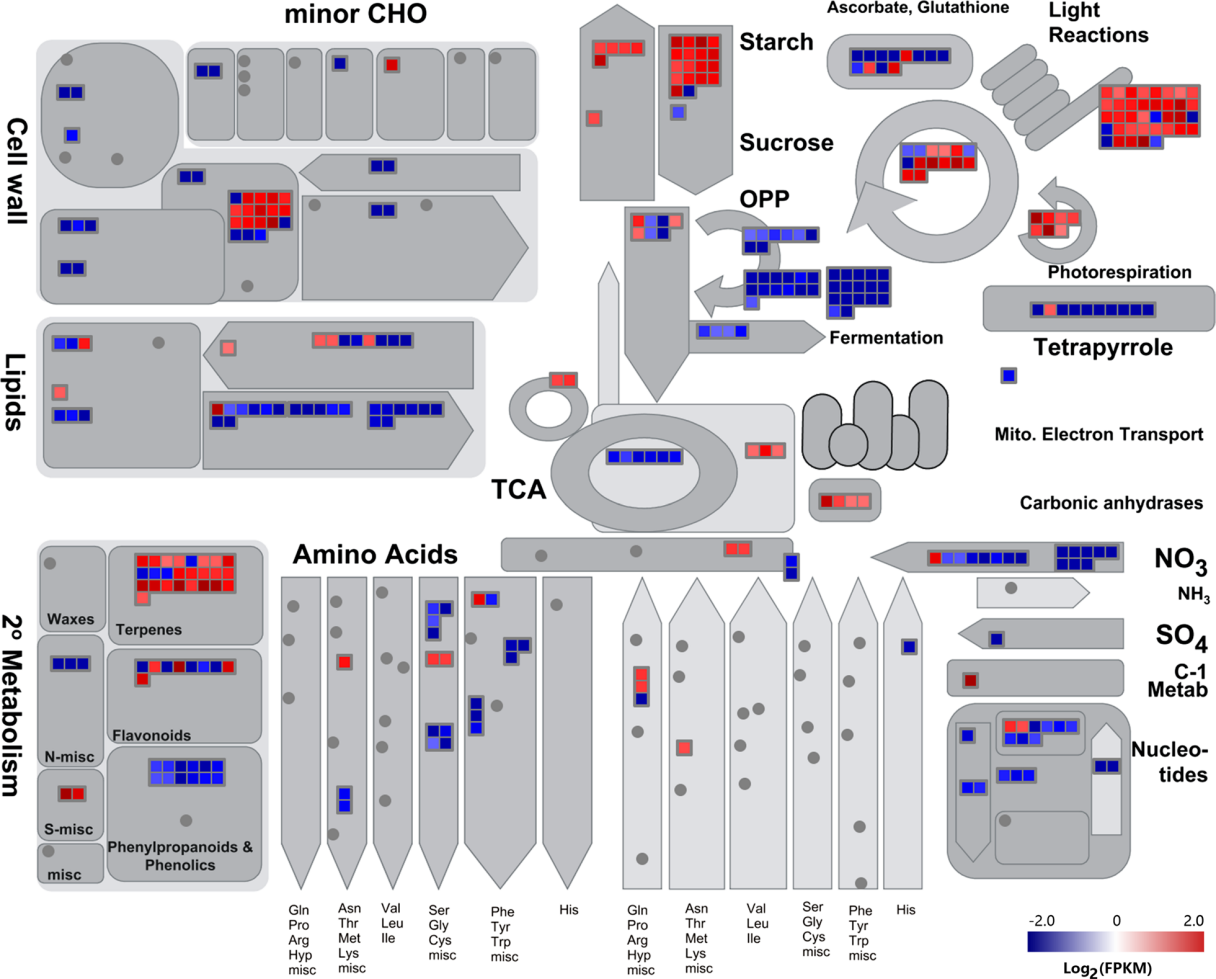
Metabolism overview in *L. aequinoctialis* during nitrogen starvation. Genes are represented by squares. The color scale corresponds to the log_2_-transformed expression ratios of 3 days versus 0 day. The log2-transformed FPKM values were imported into MapMan software to generate the overview of general metabolism at 3 days compared to 0 day. DEGs are represented by *colored squares* and grouped according to functional annotation based on MapMan ontology. The fold change of DEGs is indicated by the *scale bar*. *Red* indicates up-regulation, whereas *blue* indicates down-regulation

Seventy-two DEGs were enriched in starch and sucrose metabolism pathways, and more than 100 DEGs related to the starch pathways were differentially expressed during nitrogen starvation. Starch biosynthesis involves a series of key enzymes including soluble starch synthase (SS), ADP-glucose pyrophosphorylase (AGP), granule bound starch synthase (GBSS), and starch-branching enzyme (SBE), whereas the degradation of starch is catalyzed by α-amylase and β-amylase [30]. Transcripts encoding AGP and SS were significantly up-regulated under nitrogen starvation (Figs. 4, 5). However, the expression of transcripts encoding GBSS and SBE under nitrogen starvation showed no significant differences from the untreated samples. In addition, the expression of genes encoding enzymes related to glycolysis, gluconeogenesis, UDP-glucose, GDP-glucose, and α-d-glucose-1P were also analyzed under nitrogen starvation (Figs. 4, 5). The expression of unigenes encoding enzymes involved in gluconeogenesis (e.g., phosphoenolpyruvate, glycerate-1,3P_2_, fructose-1,6P_2_, glucose-1P) were up-regulated, while the majority of unigenes involved in glycolysis (e.g., glycerate-3P, glyceraldehyde-3P) were down-regulated (Figs. 4, 5). Based on these results, it can be hypothesized that more glucose-1P might be partitioned into the starch and sucrose metabolic pathways for starch biosynthesis in response to nitrogen starvation.

Glucose-1P can be converted into UDP-glucose and GDP-glucose, which potentially compete with starch biosynthesis for substrates. UDP-glucose is generated from glucose-1P through UTP-glucose-1-phosphate uridylyltransferase and is further catalyzed by UDP-glucose 6-dehydrogenase to produce UDP-alpha-d-glucuronate [31]. Transcripts encoding UDP-glucose 6-dehydrogenase were found to be significantly down-regulated under nitrogen starvation (Fig. 5). In contrast, no significant changes in expression were observed for transcripts encoding enzymes in the GDP-glucose branch of the pathway. Overall, under nitrogen starvation, genes involved in starch and related pathways had been activated and led to starch accumulation.

### Pathways repressed by nitrogen starvation

We observed that a substantial number of DEGs were down-regulated under nitrogen starvation and led to significant changes in some metabolic pathways, including nitrogen metabolism, photosynthesis, amino acid biosynthesis, unsaturated fatty acid biosynthesis, and fatty acid degradation (Fig. 3). It is noteworthy that 30 DEGs were found to be significantly enriched in nitrogen metabolism pathways (Fig. 3). For example, genes encoding nitrate reductase (e.g., nas B) and ferredoxin–nitrite reductase (e.g., nir A) were down-regulated during nitrogen starvation, which indicated that the nitrite and NH_3_ contents were decreased. In addition, the expression of genes encoding glutamine synthetase (e.g., gln A) and glutamate synthase (e.g., GLT 1) were also down-regulated, which suggested that the l-glutamine (l-Gln) and l-glutamate (l-Glu) contents were also decreased under nitrogen starvation (Fig. 5).

DEGs associated with the photosynthetic pathways were significantly down-regulated during nitrogen starvation (Fig. 5). For example, expression of unigenes involved in photosystems II (e.g., Psb A, Psb E, Psb O, and Psb U) and photosynthetic electron transport (e.g., Pet E, Pet F, and Pet H) were significantly down-regulated at 3 and 7 days under nitrogen starvation compared to the 0 day control. To determine whether the level of chlorophyll content was decreased under nitrogen starvation, the contents of chlorophyll *a* and chlorophyll *b* were measured. The results indicated that the contents of chlorophyll *a* and chlorophyll *b* gradually decreased after nitrogen starvation stress was induced (Additional file 9: Figure S7). These results indicated that a progressive loss of photosynthetic pigments accompanied the decreased expression of genes in the photosynthetic pathways in duckweed during nitrogen starvation stress.

Genes encoding enzymes involved in fatty acid-related pathways were down-regulated, including genes involved in the biosynthesis of unsaturated fatty acid, alpha-linolenic acid metabolism, and fatty acid degradation (Fig. 3). These genes were down-regulated and may then lead to a decrease in the fatty acid biosynthesis.

Under nitrogen starvation, the transcript levels of genes involved in amino acid metabolism were also affected. Generally, the contents of protein were decreased, and the expression of genes involved in amino acid biosynthesis were down-regulated under nitrogen starvation. Consistent with the lower expression of transcripts, the protein content was decreased and might activate starch accumulation under nitrogen starvation (Figs. 3, 5).

### Metabolite analysis validates the effect of transcriptome changes on carbon participation

**Fig. 6:**
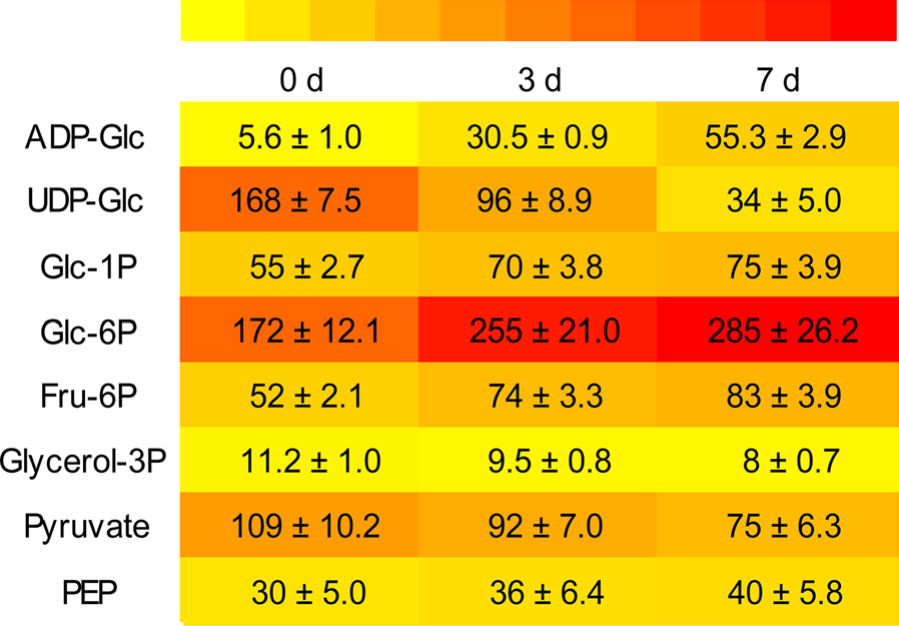
Heatmap of metabolite contents of duckweed fronds during nitrogen starvation. Fronds were collected at different time points and used for metabolite content measurement. The fold change of contents is indicated by the scale bar. *Red* indicates a higher content, while *yellow* indicates a lower content

**Fig. 7:**
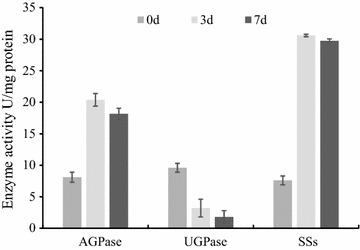
The enzyme activity of AGPase, UGPase, and SSs during nitrogen starvation at 0, 3, and 7 days. Fronds were collected at different time points and used for a starch metabolism-related enzymatic activity assay after nitrogen starvation. All data are presented as the mean of triplicate measurements ± standard deviation

The effects of the transcriptome changes discussed in the previous sections were validated using liquid chromatography–tandem mass spectrometry (LC–MS/MS) analysis of specific metabolites in *L. aequinoctialis* 6000 during nitrogen starvation. The results showed that more ADP-Glc accumulated at 3 and 7 days under nitrogen starvation compared to the 0 day control (Fig. 6). In contrast, lower levels of UDP-Glc accumulated at 3 and 7 days under nitrogen starvation compared to the samples at 0 day (Fig. 6). Furthermore, the enzyme activities of AGPase and UGPase under nitrogen starvation were determined, and the results showed that the activity of AGPase was significantly increased, while the activity of UGPase dramatically decreased under nitrogen starvation, which was consistent with the transcriptome and metabolism results (Figs. 6, 7). In addition, the contents of α-ketoglutarate and oxaloacetate, which are products of the TCA cycle, also increased during nitrogen starvation. It is notable that the contents of citrate and isocitrate also decreased during nitrogen starvation.

We also measured the metabolites of glycolysis and gluconeogenesis during nitrogen starvation. The results showed that fructose-1,6P and fructose-6P significantly increased during nitrogen starvation (Fig. 6). Thus, it can be assumed that α-ketoglutarate and oxaloacetate generated from the TCA cycle will more likely affect starch synthesis through gluconeogenesis. In addition, the levels of glucose-6P and glucose-1P were significantly decreased at 3 and 7 days under nitrogen starvation conditions compared to the 0 day control (Fig. 6). These changes in metabolite levels during nitrogen starvation are approximately consistent with the gene expression changes in the transcriptome (Figs. 3, 5). Therefore, the mRNA expression patterns induced by nitrogen starvation have an effect on the operation of related metabolic pathways. Based on these results, it can be concluded that the increase in ADP-glucose and starch contents under nitrogen starvation are a consequence of increased output from gluconeogenesis and TCA pathways, which are accompanied by the reduction in lipids and pectin biosynthesis.

## Discussion

### Overview of transcriptome dynamics of *Lemna aequinoctialis* during nitrogen starvation

Previous studies have shown that starch accumulation in duckweed can be induced by manipulating growth conditions, such as nutrient levels, temperature, pH, light intensity, and photoperiod [4]. Nutrient starvation, especially nitrogen starvation, is the most typical way to induce starch biosynthesis in duckweed [4]. Multiple changes occurred in transcripts involved with nitrogen, carbon, and energy metabolism pathways during nitrogen starvation. These changes included genes encoding proteins involved in the transfer of ammonium groups and in nitrogen assimilation and transport. In addition, these changes are also accompanied by increases in many genes associated with oxidative phosphorylation, which reflects the attempt by cells to compensate for energy deficiency during nitrogen starvation. In contrast, the transcript abundance of many genes involved in photosynthesis and protein biosynthesis was significantly decreased. In addition, the transcriptome and metabolite analysis indicated that carbon metabolism shifted from glucose synthesis to utilization and storage of starches rather than fatty acids (Fig. 5). These data defined the transcript profiles that reflect the global response to nitrogen limitation over a 7-day period in duckweed. The increased starch synthesis and storage might suggest that duckweed can serve as an important source of bioethanol feedstock, and can be utilized as a model system to study the underlying mechanisms of starch biosynthesis.

However, it is noteworthy that the higher starch accumulation under nitrogen starvation in duckweed is attained at the expense of retarded growth resulting from significant reductions in photosynthesis and fatty acid biosynthesis.

### How was starch accumulated in *L. aequinoctialis* during nitrogen starvation?

Starch is one of the major storage compounds of duckweed and usually accumulates under stressed conditions (e.g., nitrogen starvation), which is an adaptive strategy gained during the long evolutionary process to enable the plant to survive the adverse environment [32]. The starch accumulation was significantly induced in* L. aequinoctialis* under nitrogen starvation, from an initial level around 20% of dry weight to a maximum around 60% (Fig. 1A). Strikingly, it should be noted that the starch content was continuously accumulated during nitrogen starvation for up to 9 days in *L. aequinoctialis* (Fig. 1A). Similarly, a large amount of starch is usually accumulated during nitrogen starvation stress in algae. It has been shown that an increase of 41% in starch content was observed after 24 h nitrogen starvation in *C. reinhardtii*, and the starch content was almost doubled that of the control after 144 h treatment under nitrogen starvation [34]. However, algae usually first stores carbon sources as starches and then quickly converts the starches to triacylglycerol (TAG) in response to nitrogen starvation stress [33]. It has been reported that the total fatty acid levels increased by about 50% after 24 h of nitrogen starvation, and increased further to 2- and 3.8-fold after 48 and 144 h in *C. reinhardtii* [34]. In contrast, the interconversion of starch to lipids dose not seemingly occur in duckweed. Our transcriptome data are in coincidence with this notion because the expression of massive genes involved in fatty acid synthesis were significantly down-regulated during nitrogen starvation. One of the possible reasons for this down-regulation might be due to the fact that acetyl-CoA is the substrate for both fatty acid synthesis and glycolysis/gluconeogenesis. It is noteworthy that even though the total fatty acid level was decreased under nitrogen starvation, the TAG content might not change significantly because TAG only accounts for 1% (dry weight) of duckweed biomass [35]. As duckweed is capable of substantial starch accumulation compared to the other plants, it might be in great need to reduce fatty acid biosynthesis and move more substrates toward starch synthesis. This hypothesis is supported by the significant up-regulation of genes involved in glycolysis/gluconeogenesis, which might lead more acetyl-CoA to convert to glucose-1P. In this respect, total fatty acid content was decreased, and more substrates (e.g., acetyl-CoA) were then dedicated to starch synthesis under nitrogen starvation. This dedication may partially explain why duckweed has a higher capacity for starch accumulation compared to other plants. Moreover, the shift of carbon flux from amino acids to starch under nitrogen starvation in duckweed further reinforced that notion that there exists ultimate link between carbon and nitrogen metabolisms in plants.

Although several previous studies have revealed that starch accumulation is a general response to nutrient deficiency in some plants, the mechanisms underlying starch accumulation under nutrient deficiency still remain elusive. As preliminary analysis of starch biosynthesis under nitrogen starvation has already been conducted in previous transcriptome and physiological studies [36–38], we carried out a more comprehensive analysis of the gene network during nitrogen starvation in this study by taking advantage of the integrated transcriptome and metabolism analysis. The results showed that multiple changes occurred in the expression of genes involved in nitrogen, carbon, and energy metabolism during nitrogen starvation conditions. The expression of genes associated with photosynthetic machinery was significantly reduced, which was accompanied by significant decreases in the contents of chlorophyll* a* and chlorophyll* b* (Additional file 9: Figure S7). Additionally, the expression of many genes involved in protein synthesis was reduced under nitrogen starvation conditions, which indicated that protein synthesis was likely inhibited during nitrogen starvation (Fig. 5).

The bulk of the ammonium in plant cells targets the chloroplast, where almost all the enzymes necessary for the incorporation of ammonia into carbon skeletons through the GS/GOGAT pathway are located. The glutamine synthases are involved in the catalysis of glutamine formation from ammonia and glutamate. Glutamate is among one of the few key molecules that links the amino acid metabolic pathways with the carbohydrate and lipid metabolic pathways [39]. Our results indicated that DEGs encoding proteins related to Glu and Gln biosynthesis were dramatically down-regulated under nitrogen stress, which led to an influx of metabolites into carbohydrate and lipid biosynthetic pathways (Additional file 10: Figure S8).

The flux of carbon metabolism is mainly derived from the synthesis of glucose and ends in utilization and storage as starch. To obtain more starch accumulation, two types of strategies will usually be adopted: more substrates from other metabolic pathways being dedicated to starch and sucrose metabolism or a lower starch degradation rate. Metabolic profiling can provide information on the dynamic metabolic status of a living system during stress (e.g., nitrogen starvation). Thus the metabolic profiles might help to systematically decipher the affected pathways and underlying mechanisms after stress treatment and help to corroborate the transcriptome data. Previous studies on metabolism flux have dramatically contributed towards improving our understanding of resource allocation and regulation in model plant Arabidopsis [32].

**Fig. 8:**
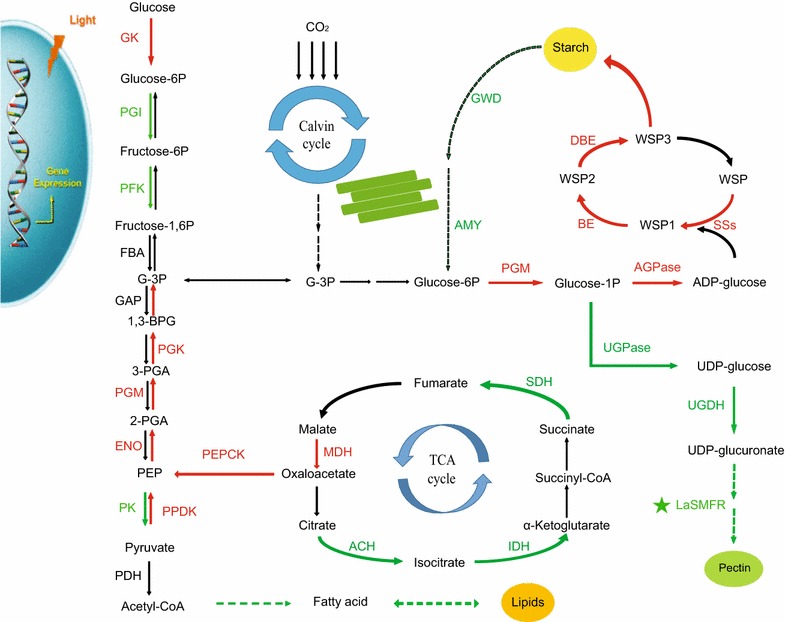
A hypothetical model of pathways related to carbohydrate metabolism during nitrogen starvation in *L. aequinoctialis*. Several biological processes as well as the related protein complex in response to nitrogen starvation are presented in the model. *The arrows* indicated the directions of catalytic reactions or transportations. *Red arrow* indicates up-regulation, while the *green arrow* indicates down-regulation

UDP-glucose plays an important role in the allocation of carbon metabolism, and it has four branches to the next metabolite, sucrose, glycogen, UDP-glucuronate, and glucose-1P. Thus, UDP-glucose is one of the important substrates for starch synthesis. In this study, DEGs involved in the UDP-glucuronate and pectin branches were significantly down-regulated during nitrogen starvation, which directed more substrates to the starch synthesis pathway (Fig. 8). Moreover, DEGs encoding the key enzymes in starch synthesis (e.g., AGPs and SSs) also had high expression levels and then catalyzed all the metabolic substrates to be efficiently transformed into starch. The transcriptome data and metabolic analysis defined the global transcript profiling and metabolic changes in response to nitrogen starvation in duckweed. The results indicated that duckweed reduced the branch to pectin biosynthesis and integrated all metabolite substrates into starch synthesis, which ultimately contributed to the higher starch accumulation during nitrogen starvation.

Taking all of the transcriptomic and metabolic changes into account, we concluded that nitrogen deprivation caused the suppression of global metabolism status and redirected the flux from the amino acid and cell wall (especially pectin) biosynthesis toward the biosynthesis of starch.

## Conclusions

Starch metabolism is a complex process triggered by changes in the levels of numerous transcripts and metabolites. Using next-generation sequencing, the transcriptome of *Lemna aequinoctialis* was analyzed during nitrogen starvation. The genes involved in nitrogen metabolism, protein and amino acid metabolism, starch and sucrose metabolism, and lipid metabolism were evaluated. The transcriptome results revealed insights into the putative molecular mechanisms underlying starch accumulation in duckweed during nitrogen starvation. Moreover, the results of metabolite analysis corroborated the transcriptome data. Overall, the results presented here describe the dynamic transcriptome changes in *L. aequinoctialis* and reveal the complex mechanisms in starch accumulation. The genes identified as being involved in starch metabolism represent excellent candidates for further genetic improvement of starch production in duckweed.

## Additional files

**Additional file 1: Table S1.** Assembly quality statistics of *L. aequinoctialis* 6000.

**Additional file 2: Table S2.** List of the whole duckweed unigenes with annotations expressed in three stages under nitrogen starvation.

**Additional file 3: Figure S1.** Statistics of the annotation of unigenes in public databases.

**Additional file 4: Figure S2.** Classification of Gene Ontology of assembled unigenes.

**Additional file 5: Figure S3.** Histogram presentation of clusters of orthologous groups (COGs) classification of unigenes.

**Additional file 6: Figure S4.** Pathway assignment based on the KEGG database.

**Additional file 7: Figure S5.** GO enrichment analysis of differentially expressed genes in *L. aequinoctialis* during nitrogen starvation.

**Additional file 8: Figure S6.** KEGG enrichment analysis of differentially expressed genes in *L. aequinoctialis* during nitrogen starvation.

**Additional file 9: Figure S7.** Decreased chlorophyll content of *L. aequinoctialis* 6000 under nitrogen starvation. The contents of chlorophyll a and chlorophyll b (mg/g) were measured in a spectrophotometric assay under nitrogen starvation conditions at 0, 3 and 7 days.

**Additional file 10: Figure S8.** Validation of the expression of candidate genes involved in glycolysis and gluconeogenesis under nitrogen starvation by quantitative Real-time PCR analysis.

## Abbreviations

ABA: abscisic acid
AGP: ADP-glucose pyrophosphorylase
GBSS: granule bound starch synthase
SBE: starch-branching enzyme
DEGs: differential expression genes
TAG: triacylglycerol
PAS: periodic Acid–Schiff
SH: Schenk and Hildebrandt
SSs: soluble starch synthase
DW: dry weight
FPKM: fragments per kilobase of transcripts per million mapped fragments
KEGG: Kyoto Encyclopedia of Genes and Genomes
NGS: next-generation sequencing
PE: paired-end
FW: fresh weight
log2FC: log2 fold change
GS: glutamine synthase
