# Supplementary figures and images for: Integrated analysis of transcriptome and metabolites reveals an essential role of metabolic flux in starch accumulation under nitrogen starvation in duckweed
Source: Biotechnol Biofuels. 2017 Jun 26;10:167. doi: 10.1186/s13068-017-0851-8 (PMC5485579; doi:10.1186/s13068-017-0851-8)

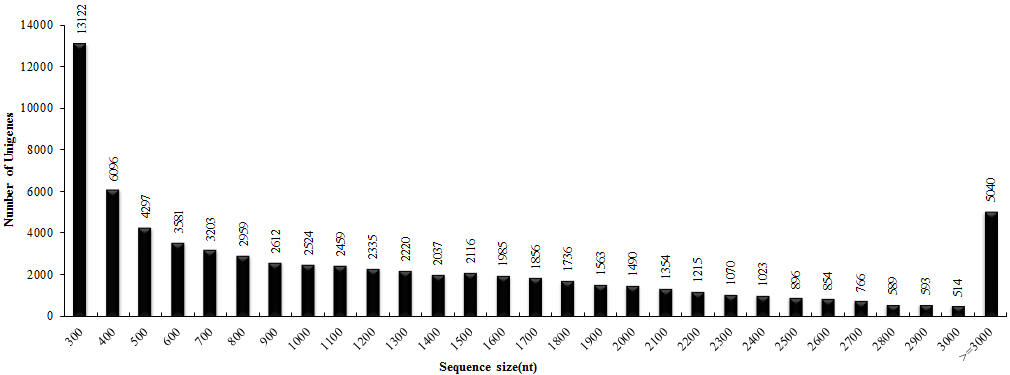

Supplement: Supplementary file 1 — Additional file 1: Table S1. Assembly quality statistics of L. aequinoctialis 6000. [file 13068_2017_851_MOESM1_ESM.tif]

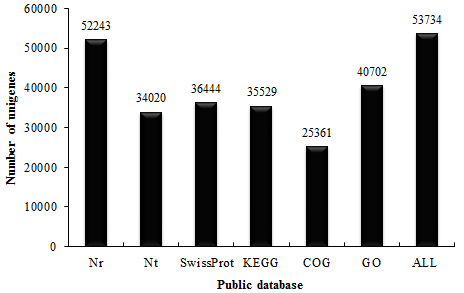

Supplement: Supplementary file 4 — Additional file 4: Figure S2. Classification of Gene Ontology of assembled unigenes. [file 13068_2017_851_MOESM4_ESM.tif]

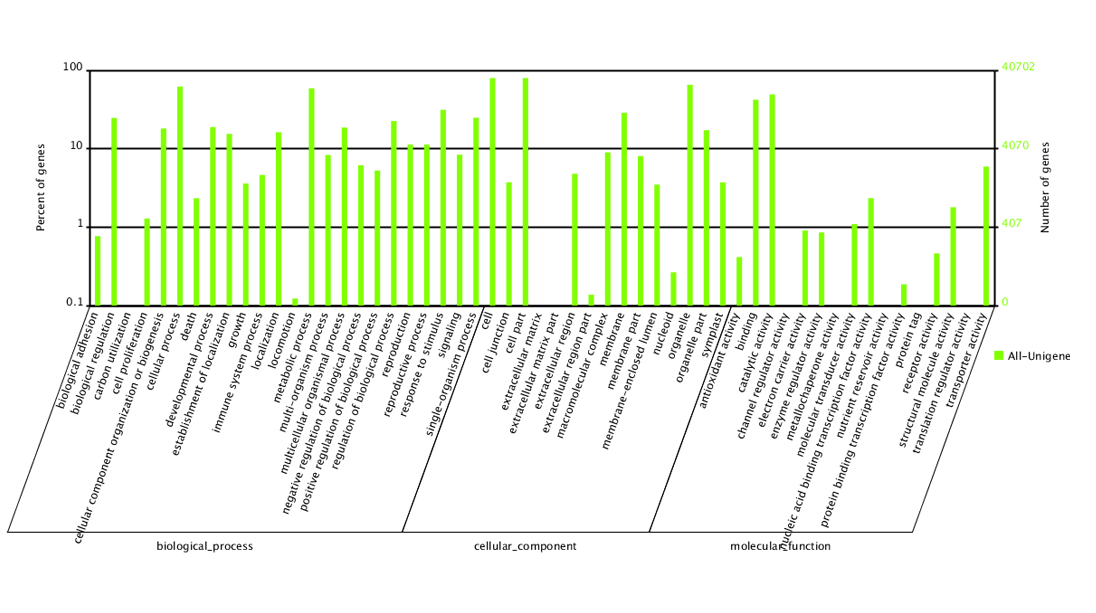

Supplement: Supplementary file 5 — Additional file 5: Figure S3. Histogram presentation of clusters of orthologous groups (COGs) classification of unigenes. [file 13068_2017_851_MOESM5_ESM.tif]

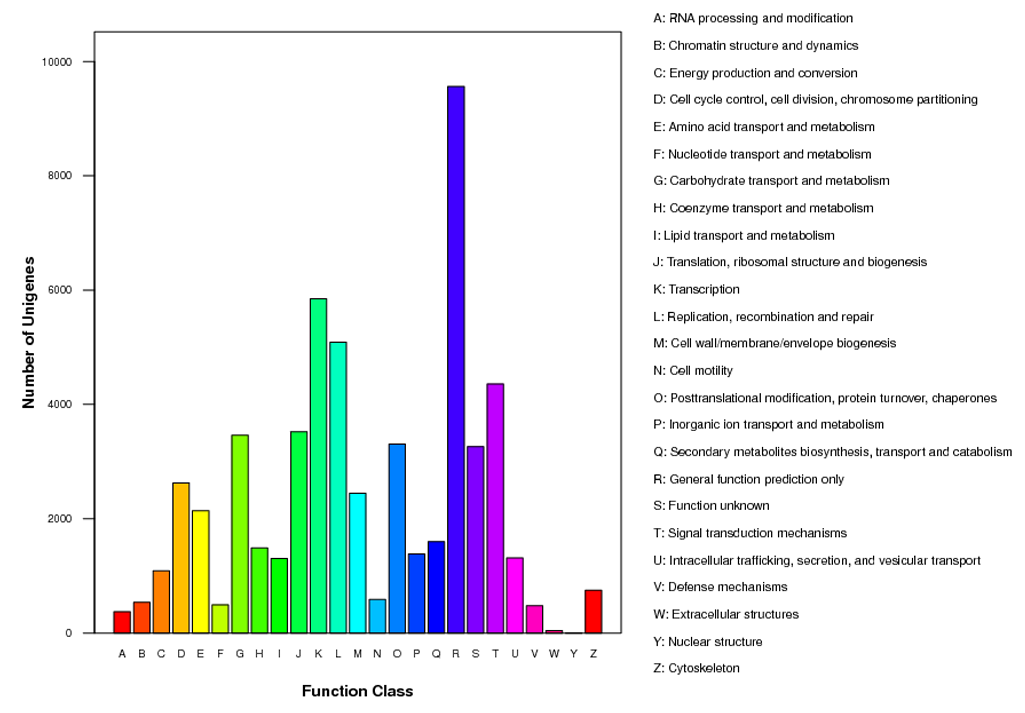

Supplement: Supplementary file 6 — Additional file 6: Figure S4. Pathway assignment based on the KEGG database. [file 13068_2017_851_MOESM6_ESM.tif]

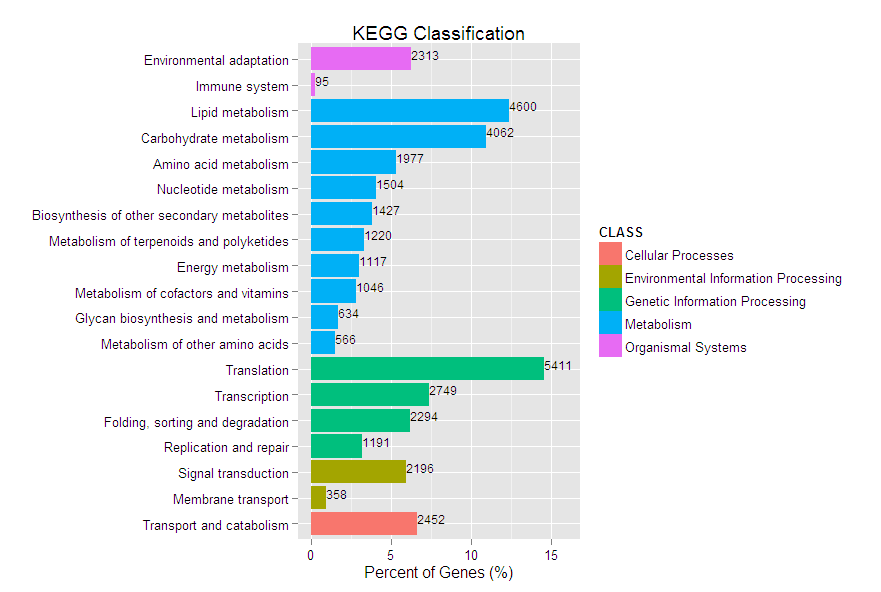

Supplement: Supplementary file 7 — Additional file 7: Figure S5. GO enrichment analysis of differentially expressed genes in L. aequinoctialis during nitrogen starvation. [file 13068_2017_851_MOESM7_ESM.tif]

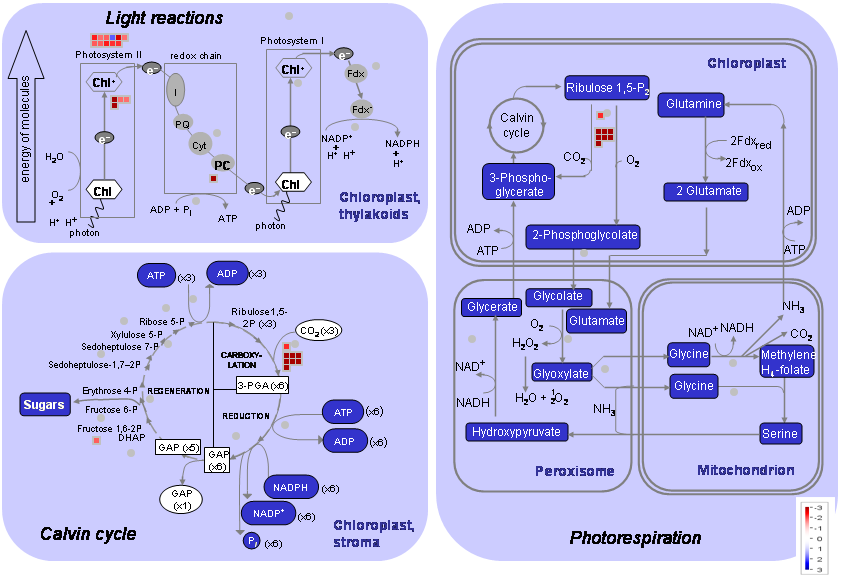

Supplement: Supplementary file 8 — Additional file 8: Figure S6. KEGG enrichment analysis of differentially expressed genes in L. aequinoctialis during nitrogen starvation. [file 13068_2017_851_MOESM8_ESM.tif]

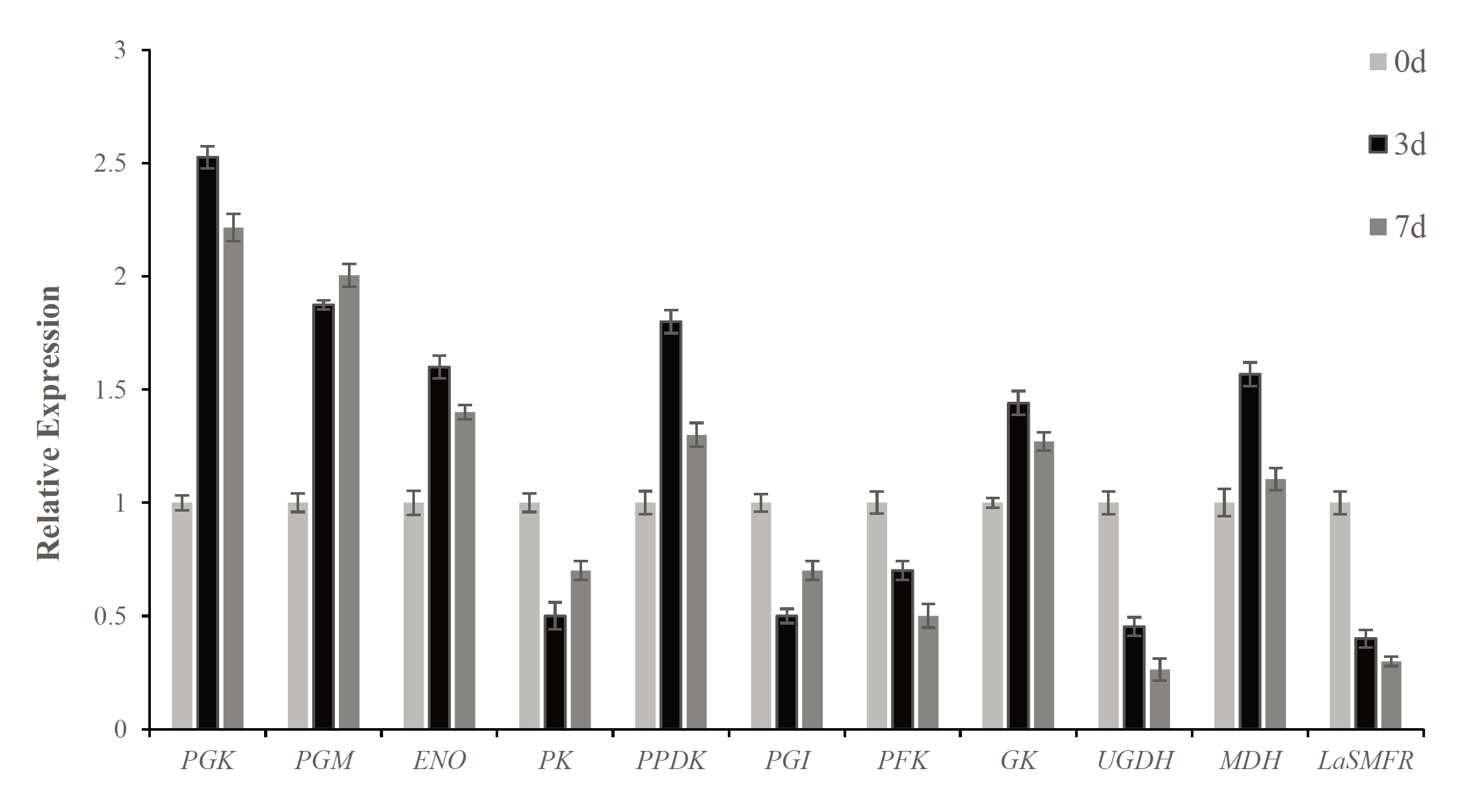

Supplement: Supplementary file 10 — Additional file 10: Figure S8. Validation of the expression of candidate genes involved in glycolysis and gluconeogenesis under nitrogen starvation by quantitative Real-time PCR analysis. [file 13068_2017_851_MOESM10_ESM.tif]
